# The histone H3K27 demethylase REF6/JMJ12 promotes thermomorphogenesis in *Arabidopsis*

**DOI:** 10.1093/nsr/nwab213

**Published:** 2021-11-25

**Authors:** Kaixuan He, Hailiang Mei, Jiaping Zhu, Qi Qiu, Xiaofeng Cao, Xian Deng

**Affiliations:** State Key Laboratory of Plant Genomics and National Center for Plant Gene Research, Institute of Genetics and Developmental Biology, Chinese Academy of Sciences, Beijing 100101, China; University of Chinese Academy of Sciences, Beijing 100049, China; Hainan Yazhou Bay Seed Lab, Sanya 572025, China; State Key Laboratory of Plant Genomics and National Center for Plant Gene Research, Institute of Genetics and Developmental Biology, Chinese Academy of Sciences, Beijing 100101, China; State Key Laboratory of Plant Genomics and National Center for Plant Gene Research, Institute of Genetics and Developmental Biology, Chinese Academy of Sciences, Beijing 100101, China; University of Chinese Academy of Sciences, Beijing 100049, China; State Key Laboratory of Plant Genomics and National Center for Plant Gene Research, Institute of Genetics and Developmental Biology, Chinese Academy of Sciences, Beijing 100101, China; State Key Laboratory of Plant Genomics and National Center for Plant Gene Research, Institute of Genetics and Developmental Biology, Chinese Academy of Sciences, Beijing 100101, China; University of Chinese Academy of Sciences, Beijing 100049, China; Center for Excellence in Molecular Plant Sciences, Chinese Academy of Sciences, Beijing 100101, China; State Key Laboratory of Plant Genomics and National Center for Plant Gene Research, Institute of Genetics and Developmental Biology, Chinese Academy of Sciences, Beijing 100101, China

**Keywords:** H3K27me3, histone demethylation, thermomorphogenesis

## Abstract

Dynamic trimethylation of histone H3 at Lys27 (H3K27me3) affects gene expression and controls plant development and environmental responses. In *Arabidopsis thaliana*, RELATIVE OF EARLY FLOWERING 6 (REF6)/JUMONJI DOMAIN-CONTAINING PROTEIN 12 demethylates H3K27me3 by recognizing a specific DNA motif. However, little is known about how REF6 activates target gene expression after recognition, especially in environmental responses. In response to warm ambient temperature, plants undergo thermomorphogenesis, which involves accelerated growth, early flowering and changes in morphology. Here we show that REF6 regulates thermomorphogenesis and cooperates with the transcription factor PHYTOCHROME INTERACTING FACTOR 4 to synergistically activate thermoresponsive genes under warm ambient temperature. The *ref6* loss-of-function mutants exhibited attenuated hypocotyl elongation at warm temperature, partially due to downregulation of *GIBBERELLIN 20-OXIDASE 2* and *BASIC HELIX-LOOP-HELIX 87*. REF6 enzymatic activity is necessary for warm ambient temperature responses. Together, our results provide direct evidence of an epigenetic modifier and a transcription factor working together to respond to the environment.

## INTRODUCTION

H3K27me3, a conserved and facultative repressive histone mark found in euchromatin, is crucial for tissue-specific gene expression and developmental regulation in multicellular eukaryotes [1,2]. The dynamic establishment and removal of H3K27me3 are mediated by polycomb repressive complexes (PRCs) and Jumonji domain-containing histone demethylases (JMJs), respectively [3–6]. In *Arabidopsis*, the main H3K27me3 demethylases are EARLY FLOWERING 6 (ELF6/JMJ 11) [7], RELATIVE OF EARLY FLOWERING 6 (REF6/JMJ12) [8], JMJ13 [9] and JMJ30 [10,11]. These demethylases synergistically restrict the H3K27me3 mark and repressive chromatin domains, and activate gene expression in a developmental-stage- and/or tissue-specific manner [6].

Despite their functional redundancy, these H3K27me3 demethylases have distinct targeting mechanisms and biological functions [12]. For instance, JMJ13 specifically recognizes H3K27me3 by hydrogen bonding and hydrophobic interactions, and acts as a temperature- and photoperiod-dependent flowering repressor [9]. ELF6 is recruited to *FLOWERING LOCUS C* by the transcription factor BRASSINAZOLE-RESISTANT1 [13], where it inhibits the floral transition [13] and prevents the transgenerational inheritance of vernalization [7]. REF6 demethylates H3K27me3 at its target loci by recognizing specific DNA motifs via its tandem zinc finger domains [8,14]; DNA methylation and chromatin status also influence REF6 targeting [15,16]. REF6 regulates multiple aspects of plant growth and development, such as flowering time [17], organ boundary formation [14], lateral root formation [18], leaf senescence [19] and seed dormancy [20]. Interestingly, REF6 is also involved in multiple hormonal regulatory pathways, such as auxin transport [18], abscisic acid catabolism [20], ethylene signaling [21] and brassinosteroid (BR) signaling [22]. However, how H3K27me3 demethylases, such as REF6, activate target gene expression, and the relationship among targeting, enzymatic activity and transcriptional activation remain unclear.

Plants have evolved diverse strategies to respond to natural environmental fluctuations [23,24]. Ambient temperature, as a major environmental signal, affects plant growth and development, geographical distribution and seasonal adaptation [25,26]. Warm ambient temperature induces dramatic morphological changes in plants, such as hypocotyl growth, petiole and root elongation, leaf hyponasty and early flowering; together, these changes are termed thermomorphogenesis [26]. Various signaling regulators and mechanisms that respond to warm temperatures have been identified [27]. For instance, external environmental cues (warm temperature, light, photoperiod, etc.) are integrated to influence hormone levels, localization and signaling for hormones such as auxin, BR and gibberellic acid (GA) [27].

The bHLH transcription factor PHYTOCHROME INTERACTING FACTOR 4 (PIF4) acts as a central regulatory hub in thermomorphogenetic responses [28]. PIF4 mediates different aspects of thermomorphogenesis by binding different targets. For instance, PIF4 binds genes involved in auxin biosynthesis (such as *YUCCA 8*, *TRYPTOPHAN AMINOTRANSFERASE OF ARABIDOPSIS 1* and *CYTOCHROME P450 FAMILY 79B*) [29,30] and BR biosynthesis (such as *DWARF4* and *BRASSINOSTEROID-6-OXIDASE 2*) [31] to mediate vegetative shoot thermomorphogenesis. PIF4 also binds the *FT* promoter to mediate flowering responses [32], and binds *SPEECHLESS* to restrict stomatal development under warm conditions [33]. In addition, warm temperature affects the expression, stability and activity of PIF4, and PIF4 integrates hormone signaling (auxin, BR and GA) in thermoresponsive regulation of growth behaviors, such as stem growth and flowering time [25,27].

Multiple regulatory mechanisms are involved in the extensive network of plant thermoresponsive growth, including transcriptional regulation, RNA metabolism and protein stability [27]. Among these, transcriptional regulation mediated by chromatin dynamics is one of the primary mechanisms [27]. Warm ambient temperatures lead to changes in occupancy of the histone variant H2A.Z [34–36] and in histone modifications (histone deacetylation [37] and histone methylation [36,38]) at genes that respond to temperature [27]. At warm temperature, the eviction of H2A.Z-containing nucleosomes at transcription start sites facilitates thermoresponsive gene expression [34,35,39]. Warm temperatures also induce H3K9 deacetylation at genes that respond to temperature, which is dependent on HISTONE DEACETYLASE 9 and POWERDRESS [37]. H3K36me3 is involved in regulating temperature-induced alternative splicing, co-transcriptional regulation and flowering time control in *Arabidopsis* [36,38]. The dynamic regulation of H3K27me3 is also essential for thermoresponsive flowering time control, which involves REF6, JMJ13 and JMJ30. REF6, together with HEAT SHOCK TRANSCRIPTION FACTOR A2, form a heritable feedback loop to induce transgenerational thermomemory for flowering [40]. JMJ13 acts as a temperature and photoperiod-dependent flowering repressor [9]. JMJ30 prevents precocious flowering caused by warm ambient temperature by removing H3K27me3 on the *FLOWERING LOCUS C* promoter [10], and functions in heat acclimation [11].

These reports revealed that histone methylation controls the balance between vegetative and reproductive growth at warm ambient temperature [41]. However, how histone methylation functions in early thermomorphogenesis and precisely regulates ambient temperature response remains elusive. Here, we demonstrate that REF6, together with the key thermomorphogenetic transcription factor PIF4, regulates plant responses to warm ambient temperature through its H3K27me3 demethylase activity. This study reveals the molecular mechanism by which REF6 participates in the response to warm ambient temperature, and demonstrates the importance of the cooperation of epigenetic factors and transcription factors in regulating gene expression and environmental responses.

## RESULTS

### REF6 promotes responses to warm ambient temperature

**Figure 1.**
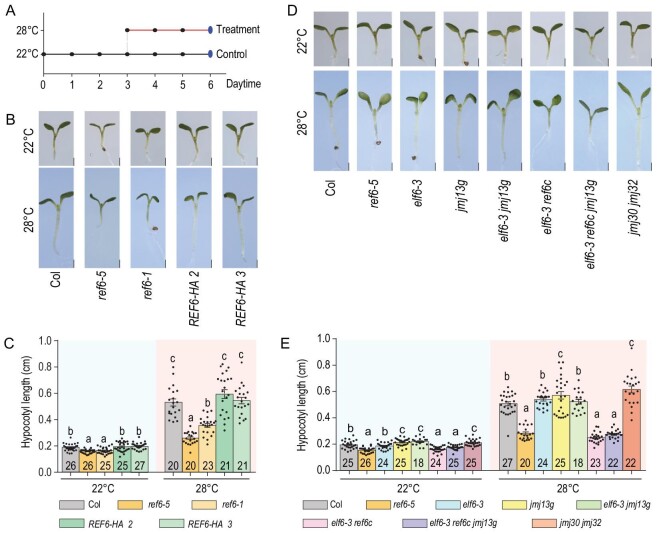
REF6 is a positive regulator of thermomorphogenesis in *Arabidopsis*. (A) Schematic representation of the temperature conditions used in this study. The dark dots represent ZT0 at each day. The time of phenotyping and collecting samples (6 days) is indicated with large blue ovals. (B–E) Phenotypic analysis. Three-day-old seedlings of the indicated genotypes grown at 22°C were transferred to 22°C or 28°C for 3 days, after which representative plants were imaged (B and D, scale bars: 1 mm) and the hypocotyl length of each plant was subsequently measured (C and E). *N* is marked in the column, and error bars depict ± s.e.m (Standard Error of Mean). Student's *t*-test was used to calculate the *P * value between the indicated genotypes, and significant differences are shown by different letters (*P* < 0.01). The dots denote individual data points.

Epigenetic regulation is highly dynamic and multiple transcription factors cooperate with epigenetic regulators to respond to environmental changes. However, little is known about how the epigenetic regulators target and function at their target genes. Our previous study showed that REF6 demethylates H3K27me3 at its target loci by recognizing specific DNA motifs, and is implicated in plant development and responses to stimuli [8,14,15]. Moreover, hypocotyl elongation in *ref6* mutants is insensitive to warm ambient temperature. We then used hypocotyl elongation as a system to investigate the function of REF6 in targeting, H3K27me3 demethylation and activation of target genes. Three-day-old seedlings grown at 22°C were transferred to 22°C or 28°C for 3 days in long-day conditions (Fig. 1A). Hypocotyl elongation of wild-type Col, *ref6* mutants (*ref6-1* weak allele and *ref6-5* null allele) and REF6 complementation lines (p*REF6::REF6-HA ref6-1*, *REF6-HA* hereafter), were recorded after 3 days of treatment at 22°C or 28°C (Fig. 1A). We found strong hypocotyl elongation responses were observed in wild-type Col seedlings at warm ambient temperature (28°C). However, although the *ref6* mutants grew normally (like the wild-type Col) at 22°C, the mutants exhibited markedly attenuated responses at 28°C compared with the wild-type seedlings (Fig. 1B and C). The attenuated hypocotyl length of *ref6* mutant at 28°C is mainly due to the reduced cell length, but not the cell number (Supplementary Fig. 1). *REF6-HA* transgenic lines rescued the attenuated hypocotyl elongation of the *ref6* mutants at 28°C (Fig. 1B and C). These results suggest that REF6 is involved in regulating thermal responses.

In addition to REF6, several other Jumonji domain-containing lysine (K)-specific demethylases, namely JMJ11/ELF6 [7,13], JMJ13 [9] and JMJ30 [10], demethylate H3K27 and function in various biological processes, such as epigenetic reprogramming [7] and temperature-dependent flowering [9,10,13]. To investigate whether these H3K27 demethylases are also involved in thermomorphogenesis, we compared the hypocotyl phenotypes of the *elf6*, *jmj13*, *elf6 ref6*, *elf6 ref6 jmj13* and *jmj30 jmj32* mutants (Fig. 1D and E). The hypocotyl lengths of these mutants were similar to those of the wild-type Col when grown at 22°C. In response to elevated ambient temperature (28°C), the *elf6 ref6* and *elf6 ref6 jmj13* mutant plants, but not the *elf6*, *jmj13* and *jmj30 jmj32* mutants, showed reduced hypocotyl elongation, similar to that of the *ref6* mutant (Fig. 1D and E). Taken together, these results indicate that REF6 is the main H3K27me3 demethylase that regulates thermomorphogenesis in *Arabidopsis*.

### Targeting of REF6 is independent of temperature changes

**Figure 2.**
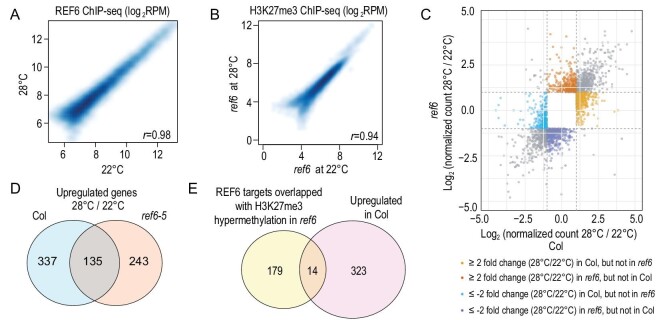
Temperature affects gene activation, but not REF6 targeting. (A) Scatterplots of normalized REF6 ChIP-seq signal intensity in log_2_ scale over all regions between 22°C and 28°C. (B) Scatterplots of normalized H3K27me3 ChIP-seq signal intensity in log_2_ scale over all regions of *ref6* mutants at 22°C and 28°C. (C) Scatterplot showing the fold change (FC) of Differential Expressed Genes (DEGs) in wild-type Col and *ref6* at 22°C and 28°C. The x and y axes represent log_2_ fold change of gene FPKM at 28°C vs. 22°C in wild-type Col and *ref6* mutant, respectively. (D) Venn diagrams showing the overlap of upregulated genes between wild-type Col and *ref6* mutants at 28°C compared with that at 22°C. (E) Venn diagrams showing the overlap between thermoresponsive upregulated genes only in wild-type Col and REF6 binding targets.

We next examined *REF6* expression and REF6 targeting activity at warm ambient temperature. The mRNA levels of *REF6*, as measured by reverse transcription-quantitative Polymerase Chain Reaction (PCR) (RT-qPCR), did not change at 22°C and 28°C (Supplementary Fig. 2), suggesting that *REF6* expression is not induced by warm ambient temperature. To test whether elevated temperature affects the recruitment of REF6 to its target genes, we profiled the genome-wide localization of REF6 in wild-type Col using chromatin immunoprecipitation coupled with high-throughput sequencing (ChIP-seq) with anti-REF6 antibody [16] at 22°C and 28°C (Supplementary Table 1). The *ref6-5* mutant was included as a negative control. Two biological replicates of REF6 ChIP-seq showed a high correlation (Pearson's correlation coefficient, *r* = 0.86 at 22°C, *r* = 0.90 at 28°C) with each other (Supplementary Fig. 3). A total of 1954 and 2136 peaks covering 1834 and 1981 genes were bound by REF6 at 22°C and 28°C, respectively. These were highly correlated (*r* = 0.98) and overlapped (Fisher's exact test, *P* < 2.2 × 10^–16^) with each other (Fig. 2A), indicating that elevated ambient temperature did not influence REF6 genome-wide targeting.

We then checked the H3K27me3 levels in wild-type Col and *ref6* mutants by H3K27me3 ChIP-seq at 22°C and 28°C (Fig. 2B, and Supplementary Fig. 3). This analysis detected 288 high-confidence hypermethylated H3K27me3 sites in *ref6* mutants at both 22°C and 28°C, 193 of which were REF6 binding targets (Supplementary Table 2). These results demonstrated that *REF6* transcript levels, REF6 targeting and REF6 enzymatic activity are temperature insensitive.

### Temperature-dependent gene activation is dependent on REF6

Because REF6 is an H3K27me3 demethylase and H3K27me3 is associated with gene repression, we next investigated the relationship between REF6 targeting and gene activation, and whether the increased H3K27me3 levels in the *ref6* mutant influence target gene expression at warm temperatures. We therefore carried out an RNA sequencing (RNA-seq) analysis, using RNA from seedlings of wild-type Col and *ref6-5* mutant treated at 22°C and 28°C (Supplementary Table 3). A total of 74 million (79%), 76 million (80%), 77 million (82%) and 76 million (82%) clean reads of highly correlated RNA-seq replicates were uniquely aligned to the *Arabidopsis* reference genome, and were mapped to 17 092, 16 981, 17 189 and 17 127 genes in the wild-type Col and *ref6-5* mutants at 22°C and 28°C, respectively Reads Per Kilobase Million (RPKM > 1) (Supplementary Table 3). RNA-seq data from three replicate samples showed high correlation (Supplementary Fig. 4).

**Figure 3.**
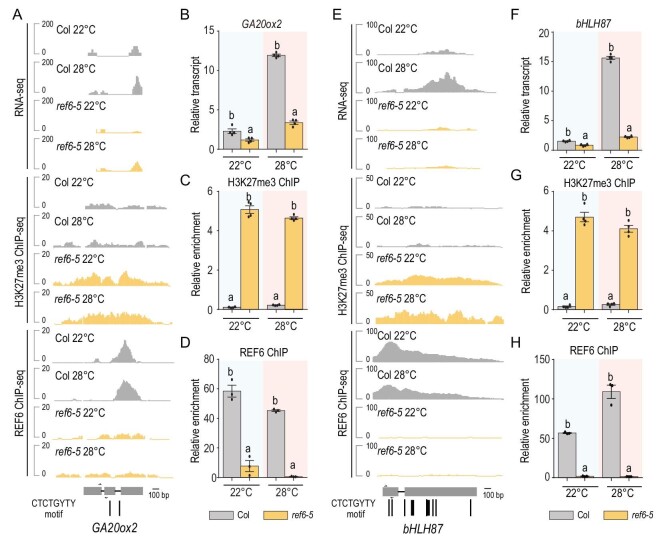
REF6 targets and promotes *GA20ox2* and *bHLH87* gene expression. (A and E) Representative genome browser view of RNA transcription, H3K27me3 and REF6 binding for (A) *GA20ox2* and (E) *bHLH87* loci in wild-type Col and *ref6-5* mutants. The locations of CTCTGYTY motifs are indicated by black bars below the gene model. The position of the primers used for ChIP-qPCR are indicated by black arrows. (B and F) The relative transcript levels of (B) *GA20ox2* and (F) *bHLH87* were validated by RT-qPCR using RNA samples of the other biological replicate. Expression was normalized to *ACTIN*. (C, D, G and H) H3K27me3 and REF6 ChIP-qPCR validation at (C and D) *GA20ox2* and (G and H) *bHLH87* using ChIP samples from another biological replicate. Expression was normalized to *ACTIN*. Enrichment was normalized to *NC4*. Data are shown as means ± s.e.m. from four and three technical replicates, respectively. Significant differences are shown by different letters (*P* < 0.01), as determined by Student's *t*-test.

Strikingly, although REF6 targeting activity is temperature insensitive, principal component analysis showed significant differences in gene expression among the wild-type Col and *ref6-5* mutants at 22°C and 28°C (Supplementary Fig. 4). For this analysis, we first calculated the fold change of transcript levels comparing 28°C to 22°C in wild-type Col and *ref6* mutants (Fig. 2C). Since REF6 is an H3K27me3 demethylase and increased H3K27me3 in *ref6* mutants leads to decreased gene expression [8], we hypothesized that the activation of REF6 target genes at increased temperature may be inhibited because of H3K27me3 hypermethylation in the *ref6* mutant. Therefore, we mainly focused on the genes that were upregulated (efficiently induced) in wild-type Col at 28°C compared with 22°C, and inefficiently induced in *ref6* mutants (yellow dots in Fig. 2C). This identified 337 genes that were upregulated (fold change ≥2) only in wild-type Col at 28°C compared with 22°C, but not in *ref6* mutants (Fig. 2D). Among these 337 genes, 44 genes were REF6 binding targets, 14 of which were H3K27me3-hypermethylated (Fig. 2E, Supplementary Table 4 and Supplementary Fig. 5). Among these 14 genes, *GIBBERELLIN 20-OXIDASE 2* (*GA20ox2*) encodes an enzyme that is involved in GA biosynthesis and temperature responses [42], and the basic helix-loop-helix transcription factor gene *BASIC HELIX-LOOP-HELIX 87* (*bHLH87*) was reported as a PIF4 downstream target in *Arabidopsis* [43]. *GA20ox2* and *bHLH87* were upregulated by elevated temperature in wild-type Col, but were inefficiently induced in *ref6-5* mutants with high levels of H3K27me3 (Fig. 3A and E). Therefore, we chose these two genes to dissect the regulatory role of REF6 in thermomorphogenesis.

Validation of REF6 binding and H3K27me3 hypermethylation in *ref6* at *GA20ox2* and *bHLH87* by ChIP and quantitative PCR (ChIP-qPCR) with an independent batch of ChIP samples yielded consistent results (Fig. 3C, D, G and H). RT-qPCR analysis also confirmed that the upregulation of *GA20ox2* and *bHLH87* in wild-type Col at warm temperature was not fully activated in the *ref6* mutant (Fig. 3B and F). These results indicated that, although warm temperature does not influence REF6 targeting and enzymatic activity, it does affect the activation of thermoresponsive genes, which are direct targets of REF6.

### The *ref6* thermomorphogenesis phenotype is at least partly due to downregulated *GA20ox2* and *bHLH87* expression

**Figure 4.**
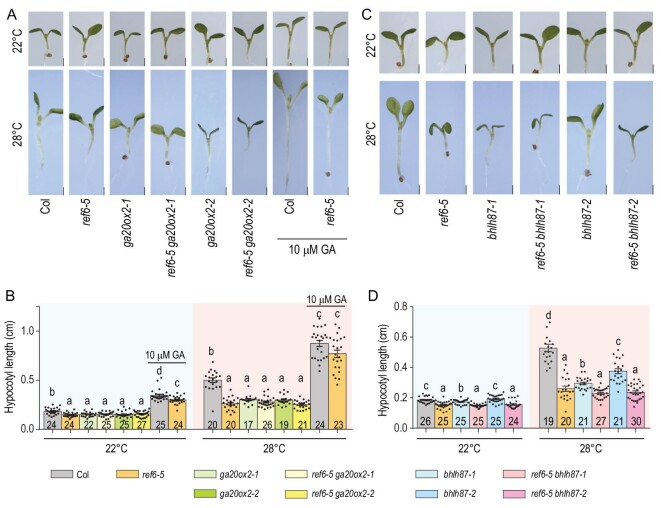
REF6 positively regulates thermomorphogenesis at least partly through *GA20ox2* and *bHLH87*. Three-day-old seedlings of the indicated genotypes grown at 22°C were transferred to 22°C or 28°C for 3 days, after which representative plants were imaged (A and C, scale bars: 1 mm) and the hypocotyl length of each plant was subsequently measured (B and D). *N* is marked in the column, and error bars depict ± s.e.m. Student's *t*-test was used to calculate the *P * value between the indicated genotypes, and significant differences are shown by different letters (*P* < 0.01). The dots denote individual data points.

To further explore whether the reduced response to warm temperature of *ref6* is associated with decreased *GA20ox2* and *bHLH87* expression, we generated *ga20ox2* and *bhlh87* Clustered Regularly Interspaced Short Palindromic Repeats/Cas9 knockout mutants in wild-type Col and the *ref6-5* mutant background (Supplementary Fig. 6). Evaluation of hypocotyl elongation at warm temperature in the wild type, double mutants and the corresponding parental lines showed that both *ga20ox2* and *bhlh87* are thermo-insensitive, similar to the *ref6-5* mutant (Fig. 4). Interestingly, in seedlings treated with 10 μM exogenous GA, the reduced hypocotyl elongation of *ref6-5* and *ga20ox2* mutants could be partially rescued at warm temperature (Fig. 4A and B, and Supplementary Fig. 7). These results indicate that REF6 regulates thermomorphogenesis at least partly through demethylating H3K27me3 and upregulating expression of *GA20ox2* and *bHLH87*.

### REF6 enzymatic activity is necessary for warm ambient temperature responses

**Figure 5.**
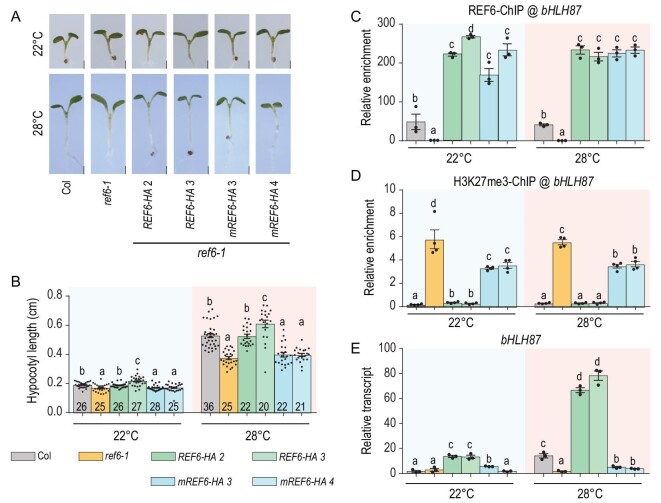
REF6 enzymatic activity is necessary for warm ambient temperature responses. (A and B) Phenotypic analysis. Three-day-old seedlings of the indicated genotypes grown at 22°C were transferred to 22°C or 28°C for 3 days, after which representative plants were imaged (A, scale bars = 1 mm) and the hypocotyl length of each plant was measured (B). *N* is marked in the column, and error bars depict ± s.e.m. Student's *t*-test was used to calculate the *P* value between the indicated genotypes, and significant differences are shown by different letters (*P* < 0.01). The dots denote individual data points. n.s. not significant. (C and D) ChIP-qPCR of (C) REF6 binding and (D) H3K27me3 levels at *bHLH87* locus using ChIP samples from the indicated plants. Three and four technical replicates were performed for each sample, respectively. (E) Transcript levels of *bHLH87* were measured by RT-qPCR and normalized to *ACTIN*. *mREF6* indicates *REF6 H246A*. Data are shown as means ± s.e.m. from three technical replicates. Significant differences are shown by different letters (*P* < 0.01), as determined by Student's *t*-test.

REF6 possesses a JmjC enzymatic domain at its N terminus, and a tandem array of C2H2 zinc finger domains at its C terminus [8]. REF6 function and genome-wide targeting involve it recognizing specific DNA motifs via its tandem zinc finger domains [14,15]. Therefore, we wondered about the relationships among targeting, enzymatic activity and transcriptional activation of REF6, in the context of warm ambient temperature. To test this, we transformed the *ref6* mutant with a construct encoding full-length REF6 with a mutation (H246A) that abolishes its enzymatic activity (p*REF6::REF6 H246A-HA*, referred to as *REF6 H246A-HA* hereafter) [8]. Transgenic lines that showed *REF6* transcript levels comparable to those of wild-type Col were chosen for further analysis (Supplementary Fig. 8). Phenotypic analysis showed that *REF6 H246A-HA ref6-1* transgenic lines did not rescue the attenuated hypocotyl elongation of the *ref6* mutants at 28°C (Fig. 5A and B), suggesting that the enzymatic activity of REF6 is essential for temperature responses.

To investigate whether REF6 enzymatic activity affects its targeting activity and transcription activation, REF6 binding, H3K27me3 status and gene activation were further validated by ChIP-qPCR and RT-qPCR of *bHLH87* and *GA20ox2* (Fig. 5C–E, and Supplementary Fig. 9). REF6 binding signals at *bHLH87* and *GA20ox2* were identical in *REF6-HA* and *REF6 H246A-HA* (Fig. 5C, and Supplementary Fig. 9A). However, H3K27 residues at these loci in *REF6 H246A-HA* plants were hypermethylated as in *ref6* (Fig. 5D, and Supplementary Fig. 9B), and the expression of *bHLH87* and *GA20ox2* was not activated in *REF6 H246A-HA* (Fig. 5E, and Supplementary Fig. 9C). Together, these results indicated that binding and enzymatic activity of REF6 are prerequisites for activation of thermoresponsive genes. Just binding of REF6 to its target loci, without enzymatic activity, cannot activate thermoresponsive gene expression, even at warm ambient temperature.

### REF6 and PIF4 are required for warm ambient temperature responses

**Figure 6.**
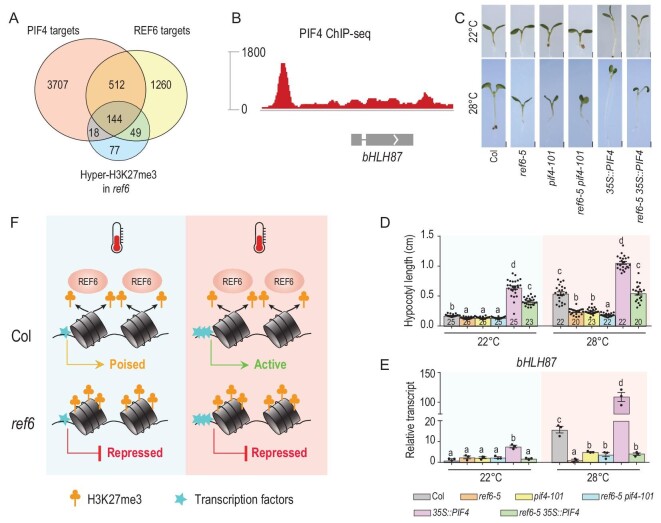
REF6 and PIF4 are required for warm ambient temperature responses. (A) Venn diagram showing the overlap of PIF4 targets, REF6 targets and H3K27me3 in the *ref6* mutant. (B) PIF4 ChIP-seq data for the *bHLH87* locus. Data are from Oh *et al*. (2012) [43]. (C and D) Phenotypic analysis. Three-day-old seedlings of the indicated genotypes grown at 22°C were transferred to 22°C or 28°C for 3 days, after which representative plants were imaged (C, scale bars: 1 mm) and the hypocotyl length of each plant was subsequently measured (D). *N* is marked in the column, and error bars depict ± s.e.m. Student's *t*-test was used to calculate the *P* value between the indicated genotypes, and significant differences are shown by different letters (*P* < 0.01). The dots denote the individual data points. (E) Expression of *bHLH87* in the indicated plants determined by RT-qPCR. Expression levels were normalized to *ACTIN*. Error bars ± s.e.m. (F) Model of how REF6 works with PIF4 to promote responses at warm ambient temperature. At 22°C, REF6 binds some thermoresponsive genes and demethylates H3K27me3, placing these genes in a poised state where they are ready for expression. At 28°C, thermo-induced transcription factors bind the thermoresponsive genes, activating their expression. However, H3K27me3 cannot be removed from these thermoresponsive genes in *ref6* mutants, so they cannot be efficiently activated at warm ambient temperature.

The bHLH transcription factor PIF4 accelerates hypocotyl growth at warm temperatures and acts as a central regulatory hub in ambient-temperature signaling, in which various signaling pathways modulate PIF4 activity, and PIF4 integrates hormone signaling in thermoresponsive regulation of growth [25,26,28]. By analyzing public ChIP-seq data sets [43], we identified 656 common targets of REF6 and PIF4, and 144 genes that have high levels of H3K27me3 in the *ref6* mutant (Fig. 6A). This set of overlapping genes includes *bHLH87* (Fig. 6B). By analyzing public transcriptome data sets [44], we compared the expression profiles of upregulated and downregulated genes between *ref6* and *pif4* mutants. We also constructed heat maps and conducted *k-*means clustering of 715 upregulated genes and 564 downregulated genes in 28°C compared with 22°C. Among these differentially expressed genes, 227 upregulated genes and 185 downregulated genes showed similar expression patterns in *ref6* and *pif4* mutants (Supplementary Fig. 10). These results indicate that REF6 and PIF4 cooperate with each other to regulate some thermoresponsive genes.

To determine whether PIF4 and REF6 directly bind to and activate *bHLH87*, we performed electrophoretic mobility shift assays (EMSAs) using DNA fragments from *bHLH87* that contain the CATATG and CTCTGTTT motifs (Supplementary Fig. 11). The DNA probes containing the CATATG motif and CTCTGTTT motif were incubated with maltose-binding-protein (MBP)-tagged PIF4 (PIF4-MBP) and Glutathione-S-transferase (GST)-tagged C-terminal REF6 (GST-REF6C). Consistent with the ChIP-seq data, PIF4 and REF6 can directly bind to *bHLH87* DNA probes (Supplementary Fig. 11). Genetic analysis showed that *pif4-101 ref6-5* plants displayed similar phenotypes to *pif4-101* plants (Fig. 6C and D). Overexpression of *PIF4* in Col enhanced the hypocotyl elongation, while the hypocotyl elongation of plants overexpressing *PIF4* in *ref6-5* was not enhanced as much as in Col at warm ambient temperature (Fig. 6C and D), indicating that REF6 and PIF4 may work together in the response to warm temperatures.

To investigate whether PIF4 and REF6 synergistically promote activation of *bHLH87* at warm temperature, we next measured the expression level of *bHLH87* in *pif4-101*, *pif4-101 ref6-5*, *35S::PIF4* and *ref6-5 35S::PIF4* plants using RT-qPCR. Compared with wild-type Col, *bHLH87* was not activated in *ref6-5*, *pif4-101* and *pif4-101 ref6-5* mutants at 22°C and 28°C (Fig. 6E). More interestingly, *bHLH87* was activated at 28°C in *35S::PIF4* plants, but not in *ref6-5 35S::PIF4* plants (Fig. 6E). These results suggested that REF6 and PIF4 work together to regulate activation of *bHLH87* at warm temperature. In this scenario, at warm ambient temperature REF6 removes H3K27me3 from *bHLH87* and PIF4 activates *bHLH87* expression (Fig. 6F).

## DISCUSSION

Epigenetic regulation is highly dynamic and diverse during development, in different cell types, and in response to changing environmental conditions. Here, we showed that the H3K27me3 demethylase REF6 regulates temperature responses in *Arabidopsis* and its targeting and enzymatic activity are essential for thermoresponsive gene activation. Loss-of-function *ref6* mutants exhibited an attenuated hypocotyl elongation phenotype (Fig. 1). Genome-wide analysis showed that REF6 directly associates with and activates the thermoresponsive genes *GA20ox2* and *bHLH87* (Figs 2–4), and REF6 enzymatic activity is essential for gene activation (Fig. 5). Interestingly, activation of *bHLH87* by warm temperature is regulated by both REF6 and the key thermoresponsive transcription factor PIF4 (Fig. 6). Together, the results of our study reveal the molecular mechanism by which REF6 participates in the response to warm ambient temperature, and demonstrate the importance of the cooperation of epigenetic factors and transcription factors in regulating gene expression and environmental responses.

Warm ambient temperatures lead to epigenetic changes, including H2A.Z nucleosomal dynamics, histone modifications and chromatin remodeling [27]. Here, we demonstrated that H3K27me3 demethylation mediated by REF6 controls plant thermal responses. At warm ambient temperatures, H2A.Z is evicted from nucleosomes, inducing thermoresponsive genes [32,34,35]. A recent study found that temperature-induced H2A.Z eviction at PIF4 targets is mediated by the histone deacetylase HDA9/PWR and the ATP-dependent chromatin remodeler INO80, and established the link between H2A.Z eviction and active transcription [37,39,45]. Since there is a strong correlation between H3K27me3 and H2A.Z enrichment [46], and the genomic targeting of INO80 requires transcription factors or chromatin regulators [45], H3K27me3 demethylation mediated by REF6 may work in concert with these factors to regulate their targets in response to thermal responses and other environmental stimuli. It will be of interest to further explore the detailed mechanism linking H3K27me3 demethylation, histone deacetylation, H2A.Z eviction, transcription factors and transcription activation.

Plants actively sense temperature and, under warm ambient temperatures, they promote the biosynthesis and localization of signaling molecules, such as auxin, BR and GA, to optimize plant morphology in a coordinated manner [27,47]. Our results found that the genes encoding key enzymes in auxin, BR and GA biosynthesis, such as *YUC8*, *BR6ox* and *GA20ox2*, respectively, are direct targets of REF6. However, only the expression of *GA20ox2*, not *BR6ox1* and *YUC8*, was induced by warm ambient temperature, which was inefficiently induced in the *ref6* mutant. This indicates that REF6 promotes thermomorphogenetic hypocotyl growth under warm temperatures by elevating thermoresponsive hormone levels (mainly GA). Thermoresponsive hormone signaling in different plant organs should be coordinated to facilitate synchronized growth and acclimation to elevated temperatures [47]. Whether and how REF6 affects hormone biosynthesis and thermomorphogenetic traits in different plant organs (such as shoots and roots), and whether they are linked with each other via inter-organ communication, will be an interesting subject for further study.

Transcription factors bind DNA in a sequence-specific manner and regulate gene expression, ensuring the appropriate level of gene expression at the right time and right place. Long-standing debates have considered the relative importance of epigenetic modifications and transcription factors in cell fate determination and other cellular processes [48,49]. At present, transcription factors and epigenetic modifiers are believed to work together to maintain normal growth and development. For instance, the transcription factor BACH1 recruits H3K4 methyltransferase complexes to maintain pluripotency in mouse embryonic stem cells [50]. In another example, the plant pioneer transcription factor LEAFY [51] and the key thermoresponsive transcription factor PIF4 [45] work with chromatin remodeling factors to open chromatin and activate transcription. Here, our detailed examination of H3K27me3 and expression levels at the *bHLH87* locus under elevated temperature suggest that *bHLH87* activation is regulated by both H3K27me3 demethylation and the thermoresponsive transcription factor PIF4, but neither REF6 nor PIF4 alone can activate *bHLH87* expression (Fig. 6). The activation of other thermoresponsive genes targeted by REF6, such as *GA20ox2*, likely requires other transcription factors that have yet to be identified. Our results provide direct evidence for the interdependent and indispensable relationship between epigenetic modifications and transcription factors with regard to synergistically promoting the expression of genes required for the response to warm temperature. This tight and indispensable control of gene activation at warm temperature is significant for the integration of temperature signals into plant morphogenesis.

How epigenetic modifiers recognize their target loci and modify the chromatin is a long-standing, fundamental question in epigenetic regulation. Our previous study showed that REF6 functions involve both self-targeting (i.e. targeting that does not require another DNA-binding protein) and DNA methylation [14,16]. Here, we demonstrated that, in addition to self-targeting, the enzymatic activity of REF6 is essential for gene activation. Expression of a mutant REF6 protein that lacks enzymatic activity (REF6 H246A) did not rescue the attenuated hypocotyl elongation of the *ref6* mutants at 28°C, but the mutation did not affect REF6 targeting to thermoresponsive target genes, suggesting that both catalytic activity and self-targeting are critical for REF6 function (Fig. 5). The targeting and enzymatic activity of a chromatin-modifying enzyme likely must be fine-tuned; therefore, additional factors, such as chromatin remodelers linking histone variants and nucleosome position, various epigenetic modifications, chromatin structure and chromatin context, may also influence its targeting and function. Further exploration of how these factors cooperatively regulate the function of a chromatin-modifying enzyme to maintain appropriate chromatin status in the genome remains an exciting topic for future research.

## METHODS

### Plant growth conditions and hypocotyl length analysis

Surface-sterilized seeds were sown on half-strength Murashige and Skoog (MS) medium supplemented with 0.8% (w/v) agar and 1% (w/v) sucrose. The plates were stratified at 4°C in darkness for 3 days, then transferred into a growth chamber (AR-22L, Percival Scientific) and germinated at 22°C for 3 days, then transferred to 28°C (treatment) or maintained at 22°C (control) at ZT0 (Zeitgeber Time) for another 3 days under a 16-h light/8-h dark cycle. The plants were grown under white fluorescent light with a fluence rate of ∼75 μmol/m^2^/sec. Seedlings from each sample were scanned using ZEISS SteREO Discovery V20, and the hypocotyl length was measured using ImageJ software (National Institutes of Health).

### Chromatin immunoprecipitation assays and library preparation

About 2.5 g of seedlings was used for ChIP assay, as previously described [14,16], using anti-REF6 antibody (custom made by Abmart) and anti-H3K27me3 antibody (Cell Signaling Technology, C36B11). The ChIP DNA was subjected to qPCR analysis or Illumina sequencing.

### ChIP-seq analysis

All sequencing reads of ChIP-seq were aligned to the *Arabidopsis thaliana* TAIR10 reference genome using Bowtie2 (version 2.2.1) [52] with default parameters after removal of the adapter sequences and low-quality bases by Cutadapt (version 1.16) and PRINSEQ (version 0.20.4) [53]. After filtering out PCR duplicates with SAMtools (version 0.1.8) [54], only uniquely mapped reads were used for downstream analysis. The first read of paired-end reads was used to generate normalized genome coverage tracks by deepTools2 (version 2.5.0.1, https://deeptools.readthedocs.io/en/develop/) with the ‘–normalizeUsing RPKM –extendReads 200 –binSize 50’ options. Then the output BigWig files were visualized using the Integrative Genomics Viewer [55]. MACS2 version 2.1.1 [56] was used to call peaks with the ‘–gsize 1.19e8 –keep-dup 1’. To identify the differentially modified regions, REF6 binding peaks and H3K27me3 peaks were merged using BEDtools (version 2.17.0) [57]. Then the signal intensity in merged peak regions was estimated by read counts per million mapped reads (RPM) after being normalized to library size. Only the regions with fold change greater than 4 between two different conditions were considered as differentially modified regions. All peaks were annotated with the priority order (promoter > exon > intron > downstream > intergenic) using ChIPseeker (version 1.17.1) [58] when a single peak spanned two genomic features.

### RNA-seq analysis

For RNA-seq data, all reads were mapped to the TAIR10 reference genome using HISAT2 (version 2.0.5) [59] with default parameters. To count the uniquely mapped reads, featureCounts (version 1.6.2) [60] was used to calculate the read count per gene and RPKM value. Then the differentially expressed genes were identified by DESeq2 (version 1.20.0) [61] with these criteria: *q*-value < 0.05, log_2_ (fold change) > 2.

## DATA AVAILABILITY

ChIP-seq and RNA-seq data from this article can be found in the Gene Expression Omnibus data library under accession number GSE181292.

## Supplementary Material

nwab213_Supplemental_Files

## References

[1] Lafos M , Kroll P, Hohenstatt ML et al. Dynamic regulation of H3K27 trimethylation during *Arabidopsis* differentiation. PLoS Genet 2011; 7: e1002040. 10.1371/journal.pgen.1002040 21490956 PMC3072373

[2] Zhang X , Clarenz O, Cokus S et al. Whole-genome analysis of histone H3 lysine 27 trimethylation in *Arabidopsis*. PLoS Biol 2007; 5: e129. 10.1371/journal.pbio.0050129 17439305 PMC1852588

[3] Lu F , Li G, Cui X et al. Comparative analysis of JmjC domain-containing proteins reveals the potential histone demethylases in *Arabidopsis* and rice. J Integr Plant Biol 2008; 50: 886–96. 10.1111/j.1744-7909.2008.00692.x 18713399

[4] Margueron R , Reinberg D. The Polycomb complex PRC2 and its mark in life. Nature 2011; 469: 343–9. 10.1038/nature09784 21248841 PMC3760771

[5] Xiao J , Wagner D. Polycomb repression in the regulation of growth and development in *Arabidopsis*. Curr Opin Plant Biol 2015; 23: 15–24. 10.1016/j.pbi.2014.10.003 25449722

[6] Yan W , Chen D, Smaczniak C et al. Dynamic and spatial restriction of Polycomb activity by plant histone demethylases. Nat Plants 2018; 4: 681–9. 10.1038/s41477-018-0219-5 30104650

[7] Crevillén P , Yang H, Cui X et al. Epigenetic reprogramming that prevents transgenerational inheritance of the vernalized state. Nature 2014; 515: 587–90. 10.1038/nature13722 25219852 PMC4247276

[8] Lu F , Cui X, Zhang S et al. *Arabidopsis* REF6 is a histone H3 lysine 27 demethylase. Nat Genet 2011; 43: 715–9. 10.1038/ng.854 21642989

[9] Zheng S , Hu H, Ren H et al. The *Arabidopsis* H3K27me3 demethylase JUMONJI 13 is a temperature and photoperiod dependent flowering repressor. Nat Commun 2019; 10: 1303. 10.1038/s41467-019-09310-x 30899015 PMC6428840

[10] Gan ES , Xu Y, Wong JY et al. Jumonji demethylases moderate precocious flowering at elevated temperature via regulation of FLC in *Arabidopsis*. Nat Commun 2014; 5: 5098. 10.1038/ncomms6098 25267112

[11] Yamaguchi N , Matsubara S, Yoshimizu K et al. H3K27me3 demethylases alter HSP22 and HSP17.6C expression in response to recurring heat in *Arabidopsis*. Nat Commun 2021; 12: 3480. 10.1038/s41467-021-23766-w 34108473 PMC8190089

[12] Deng X , Qiu Q, He K et al. The seekers: how epigenetic modifying enzymes find their hidden genomic targets in *Arabidopsis*. Curr Opin Plant Biol 2018; 45: 75–81. 10.1016/j.pbi.2018.05.006 29864678

[13] Li Z , Ou Y, Zhang Z et al. Brassinosteroid signaling recruits histone 3 lysine-27 demethylation activity to FLOWERING LOCUS C chromatin to inhibit the floral transition in *Arabidopsis*. Mol Plant 2018; 11: 1135–46. 10.1016/j.molp.2018.06.007 29969683

[14] Cui X , Lu F, Qiu Q et al. REF6 recognizes a specific DNA sequence to demethylate H3K27me3 and regulate organ boundary formation in *Arabidopsis*. Nat Genet 2016; 48: 694–9. 10.1038/ng.3556 27111035

[15] Li C , Gu L, Gao L et al. Concerted genomic targeting of H3K27 demethylase REF6 and chromatin-remodeling ATPase BRM in *Arabidopsis*. Nat Genet 2016; 48: 687–93. 10.1038/ng.3555 27111034 PMC5134324

[16] Qiu Q , Mei H, Deng X et al. DNA methylation repels targeting of *Arabidopsis* REF6. Nat Commun 2019; 10: 2063. 10.1038/s41467-019-10026-1 31048693 PMC6497721

[17] Noh B , Lee SH, Kim HJ et al. Divergent roles of a pair of homologous jumonji/zinc-finger-class transcription factor proteins in the regulation of *Arabidopsis* flowering time. Plant Cell 2004; 16: 2601–13. 10.1105/tpc.104.025353 15377760 PMC520958

[18] Wang X , Gao J, Gao S et al. REF6 promotes lateral root formation through de-repression of PIN1/3/7 genes. J Integr Plant Biol 2019; 61: 383–7. 10.1111/jipb.12726 30267471

[19] Wang X , Gao J, Gao S et al. The H3K27me3 demethylase REF6 promotes leaf senescence through directly activating major senescence regulatory and functional genes in *Arabidopsis*. PLoS Genet 2019; 15: e1008068. 10.1371/journal.pgen.1008068 30969965 PMC6457497

[20] Chen H , Tong J, Fu W et al. The H3K27me3 demethylase RELATIVE OF EARLY FLOWERING6 suppresses seed dormancy by inducing abscisic acid catabolism. Plant Physiol 2020; 184: 1969–78. 10.1104/pp.20.01255 33037128 PMC7723082

[21] Zander M , Willige BC, He Y et al. Epigenetic silencing of a multifunctional plant stress regulator. Elife 2019; 8: e47835. 10.7554/eLife.47835 31418686 PMC6739875

[22] Yu X , Li L, Li L et al. Modulation of brassinosteroid-regulated gene expression by Jumonji domain-containing proteins ELF6 and REF6 in *Arabidopsis*. Proc Natl Acad Sci USA 2008; 105: 7618–23. 10.1073/pnas.0802254105 18467490 PMC2396691

[23] Zhao C , Zhang H, Song C et al. Mechanisms of plant responses and adaptation to soil salinity. The Innovation 2020; 1: 100017. 10.1016/j.xinn.2020.100017 34557705 PMC8454569

[24] Mirouze M , Paszkowski J. Epigenetic contribution to stress adaptation in plants. Curr Opin Plant Biol 2011; 14: 267–74. 10.1016/j.pbi.2011.03.004 21450514

[25] Li B , Gao K, Ren H et al. Molecular mechanisms governing plant responses to high temperatures. J Integr Plant Biol 2018; 60: 757–79. 10.1111/jipb.12701 30030890

[26] Quint M , Delker C, Franklin KA et al. Molecular and genetic control of plant thermomorphogenesis. Nat Plants 2016; 2: 15190. 10.1038/nplants.2015.190 27250752

[27] Casal JJ , Balasubramanian S. Thermomorphogenesis. Annu Rev Plant Biol 2019; 70: 321–46. 10.1146/annurev-arplant-050718-095919 30786235

[28] Koini MA , Alvey L, Allen T et al. High temperature-mediated adaptations in plant architecture require the bHLH transcription factor PIF4. Curr Biol 2009; 19: 408–13. 10.1016/j.cub.2009.01.046 19249207

[29] Sun J , Qi L, Li Y et al. PIF4-mediated activation of YUCCA8 expression integrates temperature into the auxin pathway in regulating *arabidopsis* hypocotyl growth. PLoS Genet 2012; 8: e1002594. 10.1371/journal.pgen.1002594 22479194 PMC3315464

[30] Franklin KA , Lee SH, Patel D et al. Phytochrome-interacting factor 4 (PIF4) regulates auxin biosynthesis at high temperature. Proc Natl Acad Sci USA 2011; 108: 20231–5. 10.1073/pnas.1110682108 22123947 PMC3250122

[31] Wei Z , Yuan T, Tarkowska D et al. Brassinosteroid biosynthesis is modulated via a transcription factor cascade of COG1, PIF4, and PIF5. Plant Physiol 2017; 174: 1260–73. 10.1104/pp.16.01778 28438793 PMC5462011

[32] Kumar SV , Lucyshyn D, Jaeger KE et al. Transcription factor PIF4 controls the thermosensory activation of flowering. Nature 2012; 484: 242–5. 10.1038/nature10928 22437497 PMC4972390

[33] Lau OS , Song Z, Zhou Z et al. Direct control of SPEECHLESS by PIF4 in the high-temperature response of stomatal development. Curr Biol 2018; 28: 1273–80. 10.1016/j.cub.2018.02.054 29628371 PMC5931714

[34] Kumar SV , Wigge PA. H2A.Z-containing nucleosomes mediate the thermosensory response in *Arabidopsis*. Cell 2010; 140: 136–47. 10.1016/j.cell.2009.11.006 20079334

[35] Cortijo S , Charoensawan V, Brestovitsky A et al. Transcriptional regulation of the ambient temperature response by H2A.Z nucleosomes and HSF1 transcription factors in *Arabidopsis*. Mol Plant 2017; 10: 1258–73. 10.1016/j.molp.2017.08.014 28893714 PMC6175055

[36] Sidaway-Lee K , Costa MJ, Rand DA et al. Direct measurement of transcription rates reveals multiple mechanisms for configuration of the *Arabidopsis* ambient temperature response. Genome Biol 2014; 15: R45. 10.1186/gb-2014-15-3-r45 24580780 PMC4053849

[37] Tasset C , Singh Yadav A, Sureshkumar S et al. POWERDRESS-mediated histone deacetylation is essential for thermomorphogenesis in *Arabidopsis* thaliana. PLoS Genet 2018; 14: e1007280. 10.1371/journal.pgen.1007280 29547672 PMC5874081

[38] Pajoro A , Severing E, Angenent GC et al. Histone H3 lysine 36 methylation affects temperature-induced alternative splicing and flowering in plants. Genome Biol 2017; 18: 102. 10.1186/s13059-017-1235-x 28566089 PMC5452352

[39] van der Woude LC , Perrella G, Snoek BL et al. HISTONE DEACETYLASE 9 stimulates auxin-dependent thermomorphogenesis in *Arabidopsis* thaliana by mediating H2A.Z depletion. Proc Natl Acad Sci USA 2019; 116: 25343–54. 10.1073/pnas.1911694116 31767749 PMC6911240

[40] Liu J , Feng L, Gu X et al. An H3K27me3 demethylase-HSFA2 regulatory loop orchestrates transgenerational thermomemory in *Arabidopsis*. Cell Res 2019; 29: 379–90. 10.1038/s41422-019-0145-8 30778176 PMC6796840

[41] He K , Cao X, Deng X. Histone methylation in epigenetic regulation and temperature responses. Curr Opin Plant Biol 2021; 61: 102001. 10.1016/j.pbi.2021.102001 33508540

[42] Camut L , Regnault T, Sirlin-Josserand M et al. Root-derived GA(12) contributes to temperature-induced shoot growth in *Arabidopsis*. Nat Plants 2019; 5: 1216–21. 10.1038/s41477-019-0568-8 31819220

[43] Oh E , Zhu JY, Wang ZY. Interaction between BZR1 and PIF4 integrates brassinosteroid and environmental responses. Nat Cell Biol 2012; 14: 802–9. 10.1038/ncb2545 22820378 PMC3703456

[44] Ding L , Wang S, Song ZT et al. Two B-Box domain proteins, BBX18 and BBX23, interact with ELF3 and regulate thermomorphogenesis in *Arabidopsis*. Cell Rep 2018; 25: 1718–28. 10.1016/j.celrep.2018.10.060 30428343

[45] Xue M , Zhang H, Zhao F et al. The INO80 chromatin remodeling complex promotes thermomorphogenesis by connecting H2A.Z eviction and active transcription in *Arabidopsis*. Mol Plant 2021; 14: 1799–813. 10.1016/j.molp.2021.07.001 34242850

[46] Dai X , Bai Y, Zhao L et al. H2A.Z represses gene expression by modulating promoter nucleosome structure and enhancer histone modifications in *Arabidopsis*. Mol Plant 2018; 11: 1799–813. 10.1016/j.molp.2018.03.011 29614313

[47] Park YJ , Kim JY, Lee JH et al. External and internal reshaping of plant thermomorphogenesis. Trends Plant Sci 2021; 26: 810–21. 10.1016/j.tplants.2021.01.002 33583729

[48] Davis RL , Weintraub H, Lassar AB. Expression of a single transfected cDNA converts fibroblasts to myoblasts. Cell 1987; 51: 987–1000. 10.1016/0092-8674(87)90585-X 3690668

[49] Matoba S , Liu Y, Lu F et al. Embryonic development following somatic cell nuclear transfer impeded by persisting histone methylation. Cell 2014; 159: 884–95. 10.1016/j.cell.2014.09.055 25417163 PMC4243038

[50] Niu C , Wang S, Guo J et al. BACH1 recruits NANOG and histone H3 lysine 4 methyltransferase MLL/SET1 complexes to regulate enhancer-promoter activity and maintains pluripotency. Nucleic Acids Res 2021; 49: 1972–86. 10.1093/nar/gkab034 33503260 PMC7913776

[51] Jin R , Klasfeld S, Zhu Y et al. LEAFY is a pioneer transcription factor and licenses cell reprogramming to floral fate. Nat Commun 2021; 12: 626. 10.1038/s41467-020-20883-w 33504790 PMC7840934

[52] Langmead B , Salzberg SL. Fast gapped-read alignment with Bowtie 2. Nat Methods 2012; 9: 357–9. 10.1038/nmeth.1923 22388286 PMC3322381

[53] Schmieder R , Edwards R. Quality control and preprocessing of metagenomic datasets. Bioinformatics 2011; 27: 863–4. 10.1093/bioinformatics/btr026 21278185 PMC3051327

[54] Li H , Handsaker B, Wysoker A et al. The sequence alignment/map format and SAMtools. Bioinformatics 2009; 25: 2078–9. 10.1093/bioinformatics/btp352 19505943 PMC2723002

[55] Robinson JT , Thorvaldsdottir H, Winckler W et al. Integrative genomics viewer. Nat Biotechnol 2011; 29: 24–6. 10.1038/nbt.1754 21221095 PMC3346182

[56] Zhang Y , Liu T, Meyer CA et al. Model-based analysis of ChIP-Seq (MACS). Genome Biol 2008; 9: R137. 10.1186/gb-2008-9-9-r137 18798982 PMC2592715

[57] Quinlan AR , Hall IM. BEDTools: a flexible suite of utilities for comparing genomic features. Bioinformatics 2010; 26: 841–2. 10.1093/bioinformatics/btq033 20110278 PMC2832824

[58] Yu G , Wang LG, He QY. ChIPseeker: an R/Bioconductor package for ChIP peak annotation, comparison and visualization. Bioinformatics 2015; 31: 2382–3. 10.1093/bioinformatics/btv145 25765347

[59] Kim D , Langmead B, Salzberg SL. HISAT: a fast spliced aligner with low memory requirements. Nat Methods 2015; 12: 357–60. 10.1038/nmeth.3317 25751142 PMC4655817

[60] Liao Y , Smyth GK, Shi W. featureCounts: an efficient general purpose program for assigning sequence reads to genomic features. Bioinformatics 2014; 30: 923–30. 10.1093/bioinformatics/btt656 24227677

[61] Love MI , Huber W, Anders S. Moderated estimation of fold change and dispersion for RNA-seq data with DESeq2. Genome Biol 2014; 15: 550. 10.1186/s13059-014-0550-8 25516281 PMC4302049

