# Supplementary material for: The histone H3K27 demethylase REF6/JMJ12 promotes thermomorphogenesis in Arabidopsis
Source: Natl Sci Rev. 2021 Nov 25;9(5):nwab213. doi: 10.1093/nsr/nwab213 (PMC9113104; doi:10.1093/nsr/nwab213)
Supplement: nwab213_Supplemental_Files [file nwab213_supplemental_files.zip › Supplementary_Information.docx]

**SUPPLEMENTAL INFORMATION**

**METHODS**

**Plant Materials**

All *Arabidopsis* materials used in this study were in the Columbia (Col-0) background. The *ref6-1* (SALK_001018), *ref6-5* (SALK_059549), *REF6-HA 2* and *REF6-HA 3* lines were described previously [[1-3](#_ENREF_1)]. The *pif4* [[4](#_ENREF_4)] and *35S::PIF4-HA* [[5](#_ENREF_5)] lines described previously were kindly provided by Dr. Chuanyou Li [[6](#_ENREF_6)]. *elf6 jmj13*, *elf6 jmj13 ref6* and *jmj30/32* were kindly provided by Kerstin Kaufmann [[7](#_ENREF_7)]. Independent lines of *ga20ox2* and *bhlh87* mutants were generated using the CRISPR-Cas9 system [[8](#_ENREF_8)]. The *pif4-101 ref6-5*, *35S::PIF4-HA ref6-5*, *ga20ox2 ref6-5* and *bhlh87 ref6-5* mutants were generated by genetic crossing of *ref6-5* with *pif4-101*, *35S::PIF4-HA*, *ga20ox2* and *bhlh87*, respectively.

The primers used for genotyping and sequencing are listed in **Supplemental Table 5**.

**Plant Growth Conditions**

Surface-sterilized seeds were sown on half-strength Murashige and Skoog (MS) medium supplemented with 0.8 % (w/v) agar and 1 % (w/v) sucrose. Plants treated with GA were sown on MS medium with 10 μM GA. The plates were stratified at 4 °C in darkness for 3 days. The plates were then transferred into a growth chamber (AR-22L, Percival Scientific) and germinated at 22 °C for 3 days, and then transferred to 28 °C (treatment) or maintained at 22 °C (control) at ZT0 (Zeitgeber Time) for another 3 days under a 16-h light/8-h dark cycle. The plants were grown under white fluorescent light with a fluence rate about 75 μmol/m^2^/sec.

**Analysis of Hypocotyl length**

Seedlings were grown at 22 °C for 3 days and treated at 28 °C (treatment) or maintained at 22 °C (control) for another 3 days. Seedlings from each sample were scanned using ZEISS SteREO Discovery V20, and the hypocotyl length was measured using ImageJ software (National Institutes of Health). Student’s *t*-test was performed for statistical analysis of the phenotypes. Bar charts were generated using Prism 6 (GraphPad Software).

**Plasmid Construction**

For *MBP-PIF4*, the cDNA fragment of *PIF4* was amplified by primer CF7607/CF7608 using Phanta Flash Master Mix (Vazyme #P510). Then the *PIF4* fragment was cloned into *pCAMBIA-LIC-MBP* through ligation-independent cloning as previous described [[9](#_ENREF_9)].

REF6H246A-HA was made from REF6-pEarleyGate301 by site-directed mutagenesis using a QuickChange kit (Stratagene) with primers CX5264 and CX5265 as described [[2](#_ENREF_2),[10](#_ENREF_10)]. The construct was transformed into *Agrobacterium tumefaciens* strain EHA105 and then introduced into *ref6-1* (SALK_001018) using the floral-dip method [[11](#_ENREF_11)]. The transgenic plants were selected with 100 mg/L BASTA.

Construction of the vectors for CRISPR/Cas9 editing was described previously [[8](#_ENREF_8)]. Briefly, we first phosphorylated annealed sgRNA oligomers and then introduced the oligomers into AarI-digested pKI1.1R with T4 ligase (NEB). The CRISPR/Cas9 vectors were introduced into *Agrobacterium tumefaciens* cells (strain EHA105) and then transformed into Col-0 to generate *ga20ox2* and *bhlh87*. The mutants were crossed with *ref6-5* (SALK_059549) to obtain *ref6-5 ga20ox2* and *ref6-5 bhlh87* double mutants. The primers used are listed in **Supplemental Table 5**.

**ChIP-seq and ChIP-qPCR**

For ChIP-seq, 1–2 ng DNA was used for library construction with the NEXTflex Rapid DNA-seq Prep Kit (BIOO Scientific). Primers for qPCR are listed in **Supplemental Table 5**. One intergenic region was used as a negative control (*NC4*).

**RNA Extraction and RT-qPCR Analysis**

Total RNA was extracted from seedlings with TRNzol reagent (TIANGEN) and reverse-transcribed with a HiScript III 1^st^ Strand cDNA Synthesis Kit (+gDNA wiper, Vazyme). Quantitative real-time PCR (RT-qPCR) analysis was performed on a CFX96 instrument (BIO-RAD) with EvaGreen MasterMix (abm) according to the manufacturer instructions. Three biological repeats were analyzed. RT-qPCR data were normalized to *ACTIN*. Primer sequences for RT-qPCR analysis are listed in **Supplemental Table 5**.

**Gene Ontology Analysis**

The Gene Ontology enrichment analysis was conducted using DAVID [[12](#_ENREF_12)].

**Electrophoretic mobility shift assay (EMSA)**

The GST-REF6C (a.a 1239–1360) [[1](#_ENREF_1),[3](#_ENREF_3)] and MBP-PIF4 were expressed in *E. coli* (BL21 codon plus, Stratagene) and purified using Glutathione Sepharose 4B (GE Healthcare) and Profinity IMAC Ni-Charged Resin (BIO-RAD), respectively. EMSA was performed as described with some modifications [[1](#_ENREF_1),[3](#_ENREF_3)]. Complementary oligonucleotides were annealed and 5′-end labeled with Biotin (Sangon Biotech). For each EMSA reaction, ~100 ng of expressed proteins and 1 μM biotin-labeled probes were incubated in 10 μL reaction mixture (25 mM Tris–HCl, 100 mM NaCl, 2.5 mM MgCl_2_, 0.1% CA-630, 10% glycerol, 1 μM ZnSO_4_, and 1mM DTT, pH 8.0) for one hour on ice. For competition assays, 50-fold non-labeled competitor DNA was added in the reaction 30 min before the addition of the biotin-labeled probe. The complex of protein and oligonucleotides was separated by 8% native polyacrylamide gel in 0.5 X TBE buffer (40 mM Tris–HCl, 45 mM boric acid, 1 mM EDTA, pH 8.3) at 80 V for about 80 min, and then transferred onto Hybond-N+ (GE Healthcare) membrane and crosslinked with UV-light crosslinking instrument. The biotin-labeled DNA was detected by LightShift chemiluminescence according to the standard protocol (Thermo Scientific).

**SUPPLEMENTAL FIGURE LEGEND**

**Supplemental Figure 1.** **The cell lengths of hypocotyl were reduced in *ref6*, *bhlh87*, and *ga20ox2* mutants at 28 °C**. Cell morphologies (A) and average cell length (B) for the representative seedlings of the indicated genotypes. Three-day-old seedlings of the indicated genotypes grown at 22 °C were transferred to 22 °C or 28 °C for 3 days, after which representative plants were stained Propidium iodide and imaged (**A**, Scale bars = 50 μm). Cell lengths in hypocotyls of the indicated genotypes were subsequently measured (**B**). *N* is marked in the column, and error bars depict ± s.e.m. Student’s *t*-test was used to calculate the *P* value between the indicated genotypes and significant differences were shown by different letters (*P* < 0.01). The dots denote the individual data points. The cell sizes were marked with yellow lines.

**Supplemental Figure 2. *REF6* mRNA levels did not change at** **22 °C and 28 °C. (A)** Expression of *REF6* in Col at 22 °C and 28 °C detected by RT-qPCR. Gene expression was normalized to that of the control gene *ACTIN*. Data are shown as means ± s.e.m (*n* = 4). (B) Representative genome browser view of *REF6* transcript. T-DNA insertion sites on the genomic sequences of *ref6-5* are marked on the corresponding positions.

**Supplemental Figure 3. Reproducibility of ChIP-seq.** **(A, B)** Scatterplots of normalized ChIP-seq signal intensity in log_2_ scale over all regions with anti-REF6 antibody and anti-H3K27me3 at 22 °C and 28 °C, respectively. (**C**) Scatterplots of normalized H3K27me3 ChIP-seq signal intensity in log_2_ scale over all regions of wild-type Col and *ref6* mutant at 22 °C and 28 °C.

**Supplemental Figure 4. Principal component analysis of RNA-seq data.** Principal component (PC) analysis of RNA-seq data using total RNA from seedlings of wild-type Col and *ref6* mutants treated at 22 °C and 28 °C.

**Supplemental Figure 5. Another three REF6 targets.** Representative genome browser view of transcripts, H3K27me3, and REF6 binding for the targets in wild-type Col and *ref6-5* mutants.

**Supplemental Figure** **6. *GA20ox2* and *bHLH87* gene knockout.** Mutation of *GA20ox2* and *bHLH87* in T_2_ plants. In the wild-type sequence, the underlined bases are the PAM sequence of the *GA20ox2* and *bHLH87* sgRNAs (purple characters). The red characters indicate mutated sites.

**Supplemental Figure 7. GA treatment can rescue the hypocotyl phenotype of *ga20ox2* mutants.** Three-day-old seedlings of the indicated genotypes grown at 22 °C were transferred to 28 °C for 3 days with or without GA treatment, after which the hypocotyl length of each plant was measured. *N* is marked in the column, and error bars depict ± s.e.m. Student’s *t*-test was used to calculate the *P* value between the indicated genotypes, and significant differences were shown by different letters (*P* < 0.01). The dots denote individual data points.

**Supplemental Figure 8. *REF6* expression levels in the transgenic plants.** Expression of *REF6* in *REF6-HA ref6* and *REF6H246A-HA* *ref6* plants detected by RT-qPCR. Gene expression was normalized to that of the control gene *ACTIN*. *mREF6* indicates *REF6 H246A*. Data are shown as means ± s.e.m (*n* = 4). Significant differences were shown by different letters (*P* < 0.01), as determined by Student’s *t*-test.

**Supplemental Figure 9. REF6 binding, H3K27me3, and expression levels of *GA20ox2* in transgenic plants. (A, B)** ChIP-qPCR of REF6 binding **(A)** and H3K27me3 levels **(B)** at the *GA20ox2* locus using ChIP samples from the indicated plants. Four technical replicates were performed for each sample. **(C)** Transcript levels of *GA20ox2* were measured by RT-qPCR and normalized to *ACTIN*. *mREF6* indicates *REF6 H246A*. Data are shown as means ± s.e.m. from four technical replicates. Significant differences were shown by different letters (*P* < 0.01), as determined by Student’s *t*-test.

**Supplemental Figure 10.** **Heat maps of up-regulated and down-regulated genes in *ref6* and *pif4* mutants at 22 °C and 28 °C.**

**Supplemental Figure 11. PIF4 and REF6 bind *bHLH87* *in vitro*.** EMSA showing that PIF4-MBP (A) and GST-REF6C (B) bind the indicated probes from *bHLH87* locus *in vitro*.

**Supplemental Table 1 Summary of ChIP-seq data analysis.**

**Supplemental Table 2 Overlapping genes in the REF6 binding targets and H3K27 hypermethylated genes in *ref6* mutants.**

**Supplemental Table 3 Summary of RNA-seq data analysis.**

**Supplemental Table 4 Overlapping genes in the REF6 binding targets and thermo-responsive up-regulated genes in wild-type Col at 28 °C compared with 22 °C.**

**Supplemental Table 5 Oligonucleotides used in this study.**

**REFERENCES**

1. Cui X, Lu F, Qiu Q, Zhou B, Gu L, Zhang S, Kang Y, Cui X, Ma X, Yao Q, et al.: **REF6 recognizes a specific DNA sequence to demethylate H3K27me3 and regulate organ boundary formation in Arabidopsis**. *Nat Genet* 2016, **48**:694-699.

2. Lu F, Cui X, Zhang S, Jenuwein T, Cao X: **Arabidopsis REF6 is a histone H3 lysine 27 demethylase**. *Nat Genet* 2011, **43**:715-719.

3. Qiu Q, Mei H, Deng X, He K, Wu B, Yao Q, Zhang J, Lu F, Ma J, Cao X: **DNA methylation repels targeting of Arabidopsis REF6**. *Nat Commun* 2019, **10**:2063.

4. Koini MA, Alvey L, Allen T, Tilley CA, Harberd NP, Whitelam GC, Franklin KA: **High temperature-mediated adaptations in plant architecture require the bHLH transcription factor PIF4**. *Curr Biol* 2009, **19**:408-413.

5. de Lucas M, Daviere JM, Rodriguez-Falcon M, Pontin M, Iglesias-Pedraz JM, Lorrain S, Fankhauser C, Blazquez MA, Titarenko E, Prat S: **A molecular framework for light and gibberellin control of cell elongation**. *Nature* 2008, **451**:480-484.

6. Sun J, Qi L, Li Y, Chu J, Li C: **PIF4-mediated activation of YUCCA8 expression integrates temperature into the auxin pathway in regulating arabidopsis hypocotyl growth**. *PLoS Genet* 2012, **8**:e1002594.

7. Yan W, Chen D, Smaczniak C, Engelhorn J, Liu H, Yang W, Graf A, Carles CC, Zhou DX, Kaufmann K: **Dynamic and spatial restriction of Polycomb activity by plant histone demethylases**. *Nat Plants* 2018, **4**:681-689.

8. Tsutsui H, Higashiyama T: **pKAMA-ITACHI Vectors for Highly Efficient CRISPR/Cas9-Mediated Gene Knockout in Arabidopsis thaliana**. *Plant Cell Physiol* 2017, **58**:46-56.

9. De Rybel B, van den Berg W, Lokerse A, Liao CY, van Mourik H, Moller B, Peris CL, Weijers D: **A versatile set of ligation-independent cloning vectors for functional studies in plants**. *Plant Physiol* 2011, **156**:1292-1299.

10. Earley KW, Haag JR, Pontes O, Opper K, Juehne T, Song K, Pikaard CS: **Gateway-compatible vectors for plant functional genomics and proteomics**. *Plant J* 2006, **45**:616-629.

11. Clough SJ, Bent AF: **Floral dip: a simplified method for Agrobacterium-mediated transformation of Arabidopsis thaliana**. *Plant J* 1998, **16**:735-743.

12. Huang da W, Sherman BT, Lempicki RA: **Systematic and integrative analysis of large gene lists using DAVID bioinformatics resources**. *Nat Protoc* 2009, **4**:44-57.
