# Supplementary material for: The histone H3K27 demethylase REF6/JMJ12 promotes thermomorphogenesis in Arabidopsis
Source: Natl Sci Rev. 2021 Nov 25;9(5):nwab213. doi: 10.1093/nsr/nwab213 (PMC9113104; doi:10.1093/nsr/nwab213)
Supplement: nwab213_Supplemental_Files [file nwab213_supplemental_files.zip › Supplementary_Table_1_ChIP_seq.docx]

**Supplementary Table 1 Summary of ChIP-seq data analysis.**

| Library | Library Type | Total reads | Total mapped reads (% of total) | Unique mapped reads (% of total mapped) |
| --- | --- | --- | --- | --- |
| H3K27me3_col_22_rep1 | ChIP-Seq | 20,064,259 | 11,271,018 | 7,773,093 |
| H3K27me3_col_22_rep2 | ChIP-Seq | 5,980,501 | 2,152,980 | 1,746,442 |
| H3K27me3_*ref6-5_*22_rep1 | ChIP-Seq | 10,210,834 | 5,630,754 | 3,873,296 |
| H3K27me3_*ref6-5*_22_rep2 | ChIP-Seq | 3,568,559 | 2,248,192 | 1,886,682 |
| H3K27me3_col_28_rep1 | ChIP-Seq | 17,315,056 | 11,581,929 | 7,707,637 |
| H3K27me3_col_28_rep2 | ChIP-Seq | 4,783,735 | 2,391,868 | 1,842,365 |
| H3K27me3_*ref6-5*_28_rep1 | ChIP-Seq | 18,273,404 | 8,562,278 | 5,823,258 |
| H3K27me3_*ref6-5*_28_rep2 | ChIP-Seq | 4,184,813 | 2,024,194 | 1,607,545 |
| REF6_ChIP_22_rep1 | ChIP-Seq | 8,591,747 | 5,878,032 | 5,058,851 |
| REF6_ChIP_22_rep2 | ChIP-Seq | 14,926,492 | 3,134,563 | 1,932,049 |
| REF6_ChIP_28_rep1 | ChIP-Seq | 6,429,252 | 4,080,267 | 3,537,729 |
| REF6_ChIP_28_rep2 | ChIP-Seq | 16,737,339 | 8,368,670 | 5,476,317 |
