# Supplementary material for: The histone H3K27 demethylase REF6/JMJ12 promotes thermomorphogenesis in Arabidopsis
Source: Natl Sci Rev. 2021 Nov 25;9(5):nwab213. doi: 10.1093/nsr/nwab213 (PMC9113104; doi:10.1093/nsr/nwab213)
Supplement: nwab213_Supplemental_Files [file nwab213_supplemental_files.zip › Supplementary_Table_3_RNAseq.docx]

**Supplementary Table 3 Summary of RNA-seq data analysis.**

| Library | Library Type | Total reads | Total mapped reads (% of total) | Unique mapped reads (% of total mapped) |
| --- | --- | --- | --- | --- |
| col_22_rep1 | RNA-Seq | 30,313,377 | 29,304,686 | 24,196,975 |
| col_22_rep2 | RNA-Seq | 31,190,561 | 30,057,420 | 24,906,691 |
| col_22_rep3 | RNA-Seq | 31,545,490 | 30,450,309 | 25,081,978 |
| *ref6-5*_22_rep1 | RNA-Seq | 31,187,673 | 30,009,917 | 24,747,908 |
| *ref6-5*_22_rep2 | RNA-Seq | 31,747,843 | 30,397,117 | 25,675,235 |
| *ref6-5*_22_rep3 | RNA-Seq | 31,640,029 | 30,414,640 | 25,744,272 |
| col_28_rep1 | RNA-Seq | 30,654,890 | 29,444,728 | 25,059,258 |
| col_28_rep2 | RNA-Seq | 30,728,030 | 29,553,995 | 25,228,690 |
| col_28_rep3 | RNA-Seq | 31,798,014 | 30,602,860 | 26,232,414 |
| *ref6-5*_28_rep1 | RNA-Seq | 30,761,333 | 29,666,412 | 25,319,552 |
| *ref6-5*_28_rep2 | RNA-Seq | 30,671,509 | 29,522,593 | 25,186,876 |
| *ref6-5*_28_rep3 | RNA-Seq | 30,710,852 | 29,614,733 | 25,345,293 |
