# Supplementary material for: The histone H3K27 demethylase REF6/JMJ12 promotes thermomorphogenesis in Arabidopsis
Source: Natl Sci Rev. 2021 Nov 25;9(5):nwab213. doi: 10.1093/nsr/nwab213 (PMC9113104; doi:10.1093/nsr/nwab213)
Supplement: nwab213_Supplemental_Files [file nwab213_supplemental_files.zip › Supplementary_Table_5_primer.docx]

**Supplemental Table 5 Oligonucleotides used in this study.**

**Primers for genotyping and sequencing**

| ***ref6-1* LP** | TCATATACAAGGCGTTCGGTC |
| --- | --- |
| ***ref6-1* RP** | CAGTTGCAACTCTGGAGAAGG |
| ***ref6-5* LP** | CTGGCTTTTGAGGAGGTTGTTAGGG |
| ***ref6-5* RP** | CTGAGGAACTCTGTGGGAGAAGGG |
| ***bHLH87* F** | GAGTATGAATAGTAGGAGGGGTGTATAGG |
| ***bHLH87* R** | GACTGTAATTAACCTCTTGCGGCTC |
| ***GA20ox2* F** | CATGCATGTATACCAAGCACATG |
| ***GA20ox2* R** | GGTTTACGCCTAAACTTAAGCCC |

**Primers for plasmid construction**

| **CF7607** | CTGTACTTCCAATCCAAT ATGGAACACCAAGGTTGG | *MBP-PIF4* |
| --- | --- | --- |
| **CF7608** | CCGTTATCCACTTCCAAT GTGGTCCAAACGAGAACCG |  |
| **CX5264** | GTTTAGTTGGTTTGCTTGGGCTGTGGAGGACCATGACCTT | *REF6 H246A* |
| **CX5265** | AAGGTCATGGTCCTCCACAGCCCAAGCAAACCAACTAAAC |  |
| **CF6184** | ATTGGTTCGTGTTCTTTCTCTGC | *sgRNA1* |
| **CF6185** | AAACGCAGAGAAAGAACACGAAC |  |
| **CF6190** | ATTGATTGGAGGTTAAGAAGAGA | *sgRNA2* |
| **CF6191** | AAACTCTCTTCTTAACCTCCAAT |  |
| **CF2436** | ATTGAATAGCAACAACACCTCGA | *sgRNA3* |
| **CF2437** | AAACTCGAGGTGTTGTTGCTATT |  |

**Primers for RT-qPCR**

| **CF2315** | GGTGTATAGGGCTTTCAAGTCTT | *bHLH87* |
| --- | --- | --- |
| **CF2316** | CAGAAAGAAGAAATGCTCAAAAAGG |  |
| **CF2317** | CATTAGGACAAGAGTTCGAGCAG | *GA20ox2* |
| **CF2318** | GGAGGATAATGATTGAGCCTCA |  |
| **CF7318** | TGAGTCAGAAGATAATGGCG | *REF6* |
| **CF7319** | CTATTCGTTTGGCTGTTGAC |  |
| **ACTIN-F** | GGCTGAGGCTGATGATATTC | *ACTIN* |
| **ACTIN-R** | CCATGATGTCTTGGCCTACC |  |

**Primers for ChIP-qPCR**

| **CF2315** | GGTGTATAGGGCTTTCAAGTCTT | *bHLH87* |
| --- | --- | --- |
| **CF2316** | CAGAAAGAAGAAATGCTCAAAAAGG |  |
| **CF2317** | CATTAGGACAAGAGTTCGAGCAG | *GA20ox2* |
| **CF2318** | GGAGGATAATGATTGAGCCTCA |  |
| **CX7520** | TGGCAGGTGTCAATATCAAGC | *NC4* |
| **CX7521** | AATAAGGCAGCGTTTGGAGTG |  |
