# Supplementary figures and images for: The histone H3K27 demethylase REF6/JMJ12 promotes thermomorphogenesis in Arabidopsis
Source: Natl Sci Rev. 2021 Nov 25;9(5):nwab213. doi: 10.1093/nsr/nwab213 (PMC9113104; doi:10.1093/nsr/nwab213)

Supplemental Figure 10

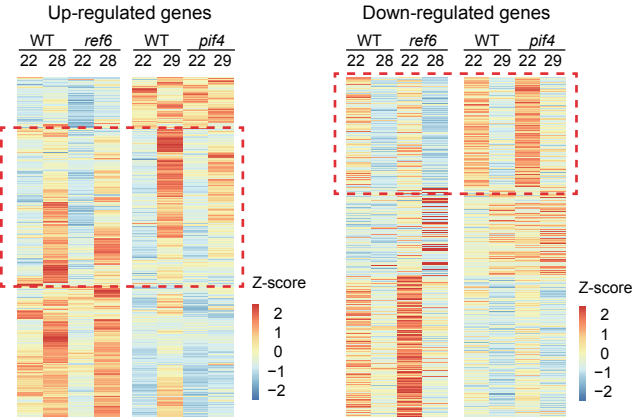

Supplement: nwab213_Supplemental_Files [file nwab213_supplemental_files.zip › A4-Sub_fig_10_140mm.pdf]

Supplemental Figure 11

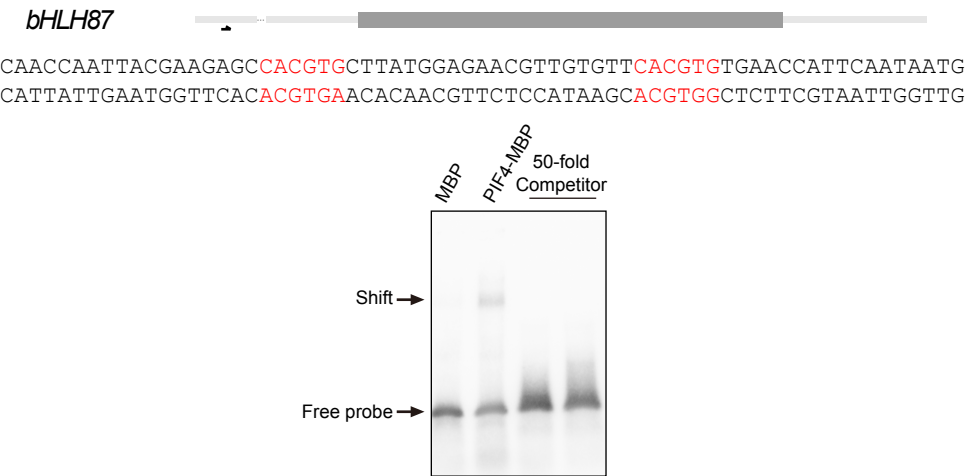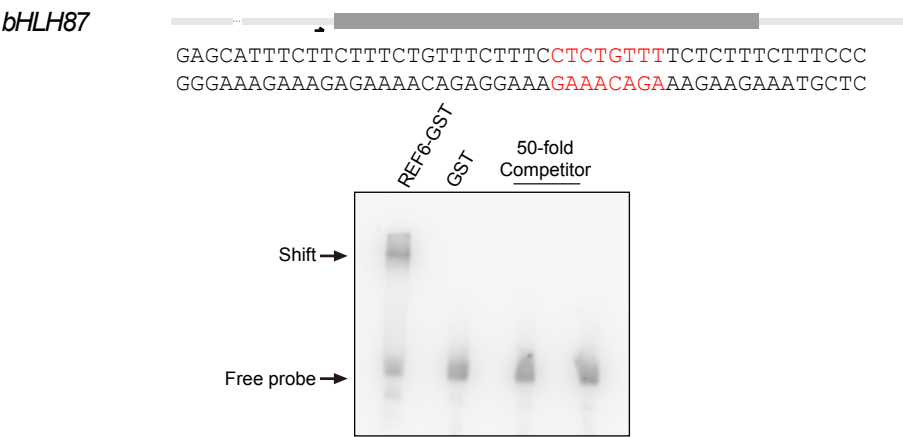

Supplement: nwab213_Supplemental_Files [file nwab213_supplemental_files.zip › A4-Sub_fig_11_140mm.pdf]

Supplemental Figure 1

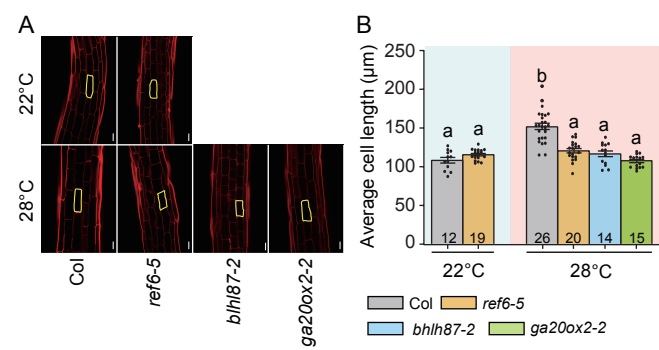

Supplement: nwab213_Supplemental_Files [file nwab213_supplemental_files.zip › A4-Sub_fig_1_96mm.pdf]

Supplemental Figure 2

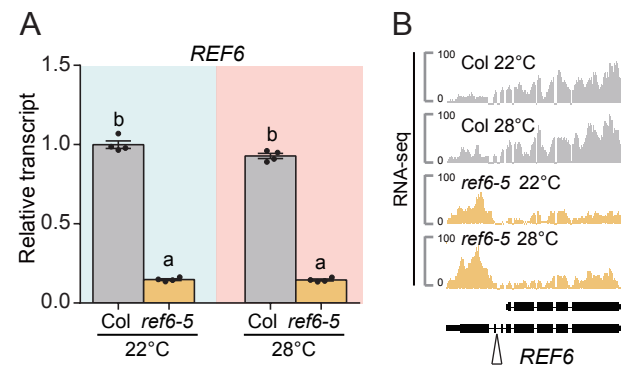

Supplement: nwab213_Supplemental_Files [file nwab213_supplemental_files.zip › A4-Sub_fig_2_96mm.pdf]

Supplemental Figure 3

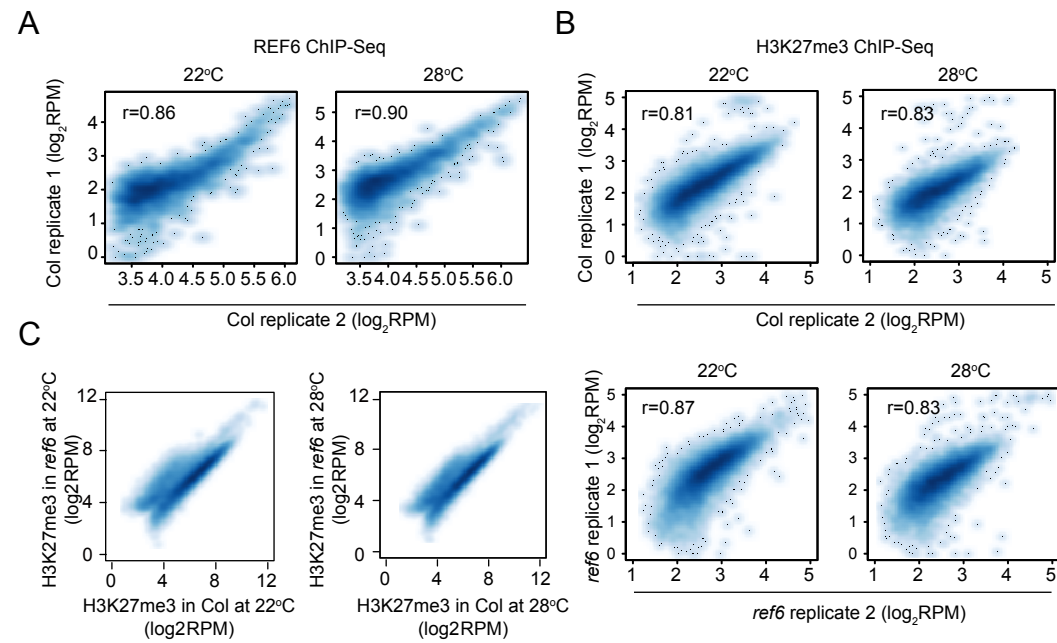

Supplement: nwab213_Supplemental_Files [file nwab213_supplemental_files.zip › A4-Sub_fig_3_140mm.pdf]

Supplemental Figure 4

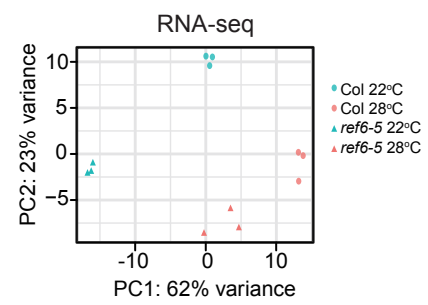

Supplement: nwab213_Supplemental_Files [file nwab213_supplemental_files.zip › A4-Sub_fig_4_60mm.pdf]

Supplemental Figure 5

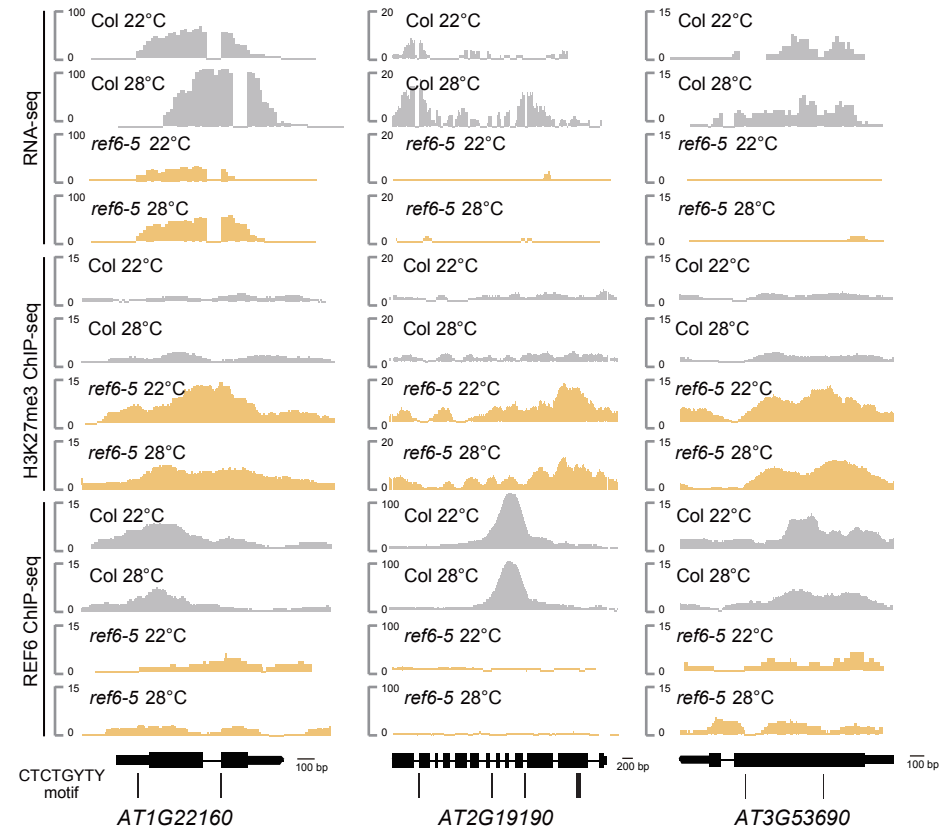

Supplement: nwab213_Supplemental_Files [file nwab213_supplemental_files.zip › A4-Sub_fig_5_140mm.pdf]

## Supplemental Figure 6

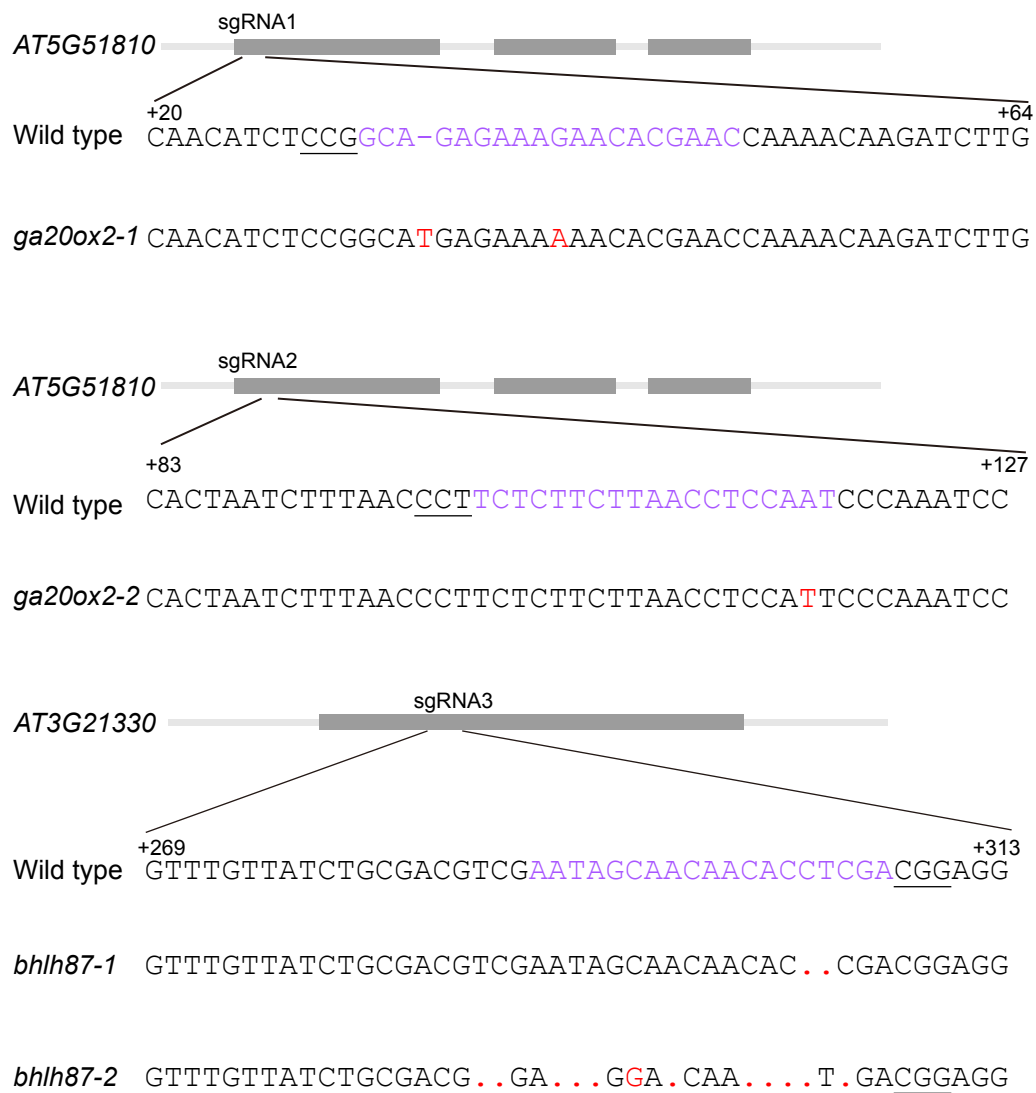

Supplement: nwab213_Supplemental_Files [file nwab213_supplemental_files.zip › A4-Sub_Fig_6_140mm.pdf]

Supplemental Figure 7

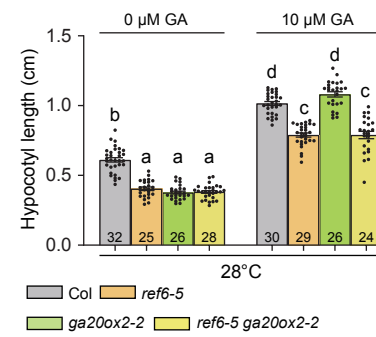

Supplement: nwab213_Supplemental_Files [file nwab213_supplemental_files.zip › A4-Sub_Fig_7_60mm.pdf]

Supplemental Figure 8

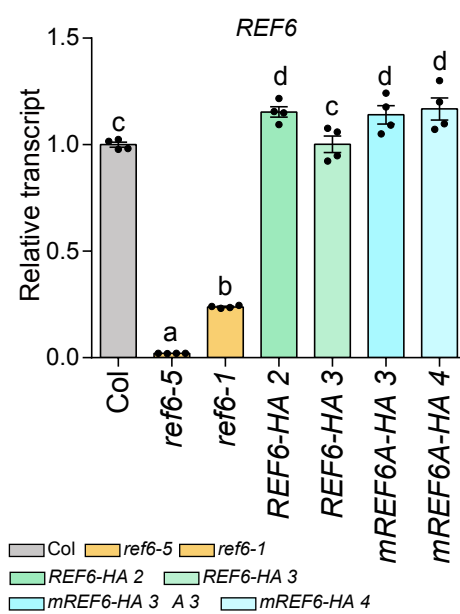

Supplement: nwab213_Supplemental_Files [file nwab213_supplemental_files.zip › A4-Sub_Fig_8_60mm.pdf]

Supplemental Figure 9

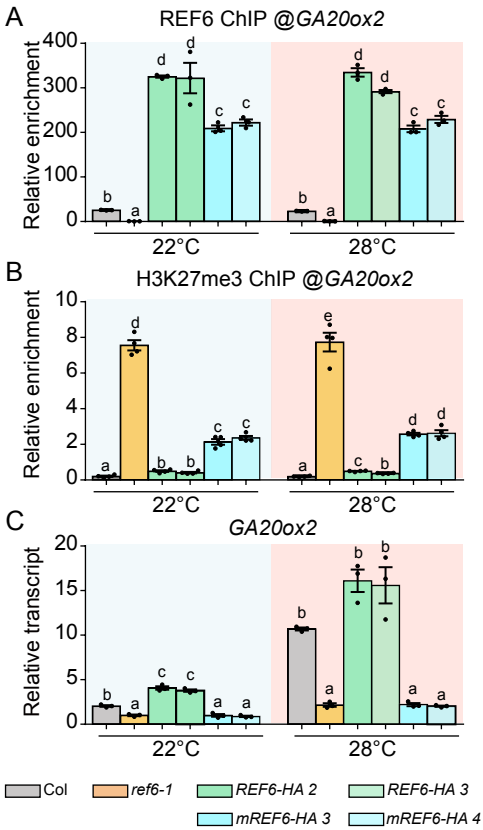

Supplement: nwab213_Supplemental_Files [file nwab213_supplemental_files.zip › A4-Sub_Fig_9_60mm.pdf]
